# Integrated Pathogen–Host Analysis of *Citrobacter braakii* SCGY-1L: Genomic Determinants and Host Transcriptional Dynamics During Infection

**DOI:** 10.3390/microorganisms13102310

**Published:** 2025-10-06

**Authors:** Zhixiu Wang, Tingting Zhou, Shaoxuan Gu, Jiaqi Yao, Suli Liu, Jiaming Mao

**Affiliations:** College of Animal Science and Technology, Yangzhou University, Yangzhou 225009, China; ztt648715244@163.com (T.Z.); w200730@126.com (S.G.); kaltist123@163.com (J.Y.); 15551957499@163.com (S.L.); 13952596198@163.com (J.M.)

**Keywords:** *Citrobacter braakii*, genomic sequencing, transcriptome profiling, host–pathogen interactions, virulence determinants, antibiotic resistance

## Abstract

*Citrobacter braakii* is an emerging opportunistic pathogen of escalating clinical significance in animal hosts, though its pathogenic mechanisms remain poorly characterized. This study isolated a *C. braakii* strain (SCGY-1L) from diseased *Siniperca chuatsi* and confirmed its identity through integrated morphological, physiological, and molecular analyses. Comprehensive genomic sequencing revealed a 5.75 Mb genome comprising one circular chromosome and two plasmids. A Circos plot was constructed to visualize the genomic architecture of strain SCGY-1L, revealing 5482 protein-coding genes, 25 tRNA genes, and 86 rRNA genes. Additionally, 738 virulence-associated genes and 366 antibiotic resistance determinants were annotated, elucidating multidrug-resistant phenotypes including insensitivity to erythromycin and penicillin. Pathogenicity assessment established an LD_50_ of 1.28 × 10^6^ CFU/mL in infected hosts, with histopathological analysis showing significant hemorrhage and necrosis in target organs (liver, spleen, kidney). Host transcriptome profiling generated 41.21 Gb of high-quality clean data, identifying 2201 differentially expressed genes post-infection (1568 up-regulated; 633 down-regulated). These were significantly enriched in phagocytosis, cytokine-mediated signaling, and inflammatory regulation pathways. These molecular insights establish *C. braakii*’s mechanistic framework for pathogenesis and host adaptation, providing critical targets for diagnostics and therapeutics against emerging *Citrobacter* infections.

## 1. Introduction

*Citrobacter* spp. are facultatively anaerobic microorganisms that are classified within the Enterobacteriaceae family. These bacteria are typically found as commensals in the gastrointestinal tract of both humans and animals. Additionally, they can be isolated from environmental sources and may serve as opportunistic pathogens in human hosts. The incidence of infections caused by *Citrobacter* species is on the rise among hospitalized individuals, and these infections frequently exhibit multidrug resistance [1,2]. Current reports on the isolation of *Citrobacter* in aquaculture and associated bacterial transmission predominantly focus on *Citrobacter freundii* [3,4]. In contrast, information regarding *Citrobacter braakii* infections in farmed aquatic animals and resulting morbidity remains relatively limited. Documented cases indicate that this pathogen can infect species such as pond-raised catfish (*Ictalurus punctatus*) [5], sea turtles (*Chelonia mydas*) [6], and Crucian Carp [7], causing diseases of varying severity. However, studies suggest that misidentification of *C. braakii* may contribute to its underreporting, ultimately leading to lower detection rates in aquaculture systems [8].

*Siniperca chuatsi*, belonging to the family Percichthyidae and order Perciformes, is natively distributed in China, Korea, and Japan and represents a high-value freshwater carnivorous species endemic to East Asia, with China dominating its global aquaculture production [9,10,11]. *S. chuatsi*, as a carnivorous and highly predatory species, exhibits broad temperature tolerance, rapid growth, and short aquaculture cycles. This species is renowned for its superior flesh quality, delicate flavor, and high nutritional value; these attributes drive substantial market demand, positioning it as a globally significant commercial aquaculture species [12,13]. It exhibits a unique feeding behavior characterized by exclusive reliance on live prey (fish/shrimp). However, recent studies in feeding ethology and metabolic physiology have enabled successful dietary domestication, establishing replicable and scalable compound feed-based aquaculture systems [14,15,16]. In intensive *S. chuatsi* aquaculture systems using either compound feeds or live bait, deterioration of rearing conditions consistently triggers outbreaks of parasitic, viral, and increasingly prevalent bacterial diseases [17]. Multiple pathogenic bacteria have been identified as causative agents in *S. chuatsi*, with zoonotic transmission risks to humans through contaminated aquatic products constituting significant public health threats [18]. *Aeromonas hydrophila* serves as the primary etiological agent of hemorrhagic septicemia, manifested by dermal hemorrhage, visceral necrosis, and systemic inflammation [19,20]. *Flavobacterium columnare* induces gill necrosis and cutaneous ulceration [21,22]. Notable secondary pathogens include *Edwardsiella tarda*, *Aeromonas veronii*, and *Streptococcus uberis*, all associated with substantially increased mortality rates in controlled infections [23,24,25].

A bacterial strain isolated from diseased *S. chuatsi* was identified as *C. braakii* through integrated morphological characterization, physiological–biochemical profiling, and 16S rRNA sequencing analysis. Whole-genome sequencing was subsequently performed, and both pathogenicity and antibiotic resistance traits of this strain were assessed. Furthermore, host transcriptomic analysis was conducted to elucidate immune response mechanisms post-infection. These results provide molecular evidence for dissecting *C. braakii* infection mechanisms and establish a theoretical foundation for clinical diagnostics and disease management strategies.

## 2. Materials and Methods

### 2.1. Bacterial Isolation

In a controlled aseptic environment, bacterial specimens were procured from diseased individuals of *S. chuatsi*. Liver tissues were inoculated onto LB agar plates utilizing an inoculation needle and subsequently incubated at 37 °C for a duration of 16 h. Morphologically distinct single colonies were selected for bacterial culture and identification. Subculturing and purification were performed through repeated streaking of different colonial morphotypes. Healthy specimens of *S. chuatsi* (average body weight: 10.26 ± 2.12 g) were sourced from a commercial aquaculture facility located in Guangzhou, Guangdong Province and laboratory-cultured under controlled conditions. Ten fish were randomly selected and confirmed negative for any pathogenic microorganisms. Prior to the initiation of experimental protocols, fish were acclimatized in aquarium tanks maintained at a temperature of 25 °C for a period of two weeks.

### 2.2. Bacterial Identification and Phylogenetic Analysis

The isolates underwent an initial characterization based on morphological assessment, which included Gram staining and scanning electron microscopy, as well as cultural characteristics, specifically examining colony morphology. The amplification of the 16S rRNA gene was executed through polymerase chain reaction (PCR) utilizing universal primers 27F (5′-AGAGTTTGATCCTGGCTCAG-3′) and 1492R (5′-GGTTACCTTGTTACGACTT-3′) [26]. The resultant sequences were aligned against the NCBI database through the application of the BLASTn tool (https://blast.ncbi.nlm.nih.gov/Blast.cgi, accessed on 10 May 2025). Phylogenetic trees were constructed employing the neighbor-joining method in MEGA version 11.0, incorporating 1000 bootstrap replicates to ensure statistical robustness. Additionally, ten virulence-associated genes (*ureF*, *ureD*, *slt-ILA*, *cfa*, *slt-LA*, *viaB*, *slt-II*, *ompX*, *ureG*, *ureE*) were identified using conventional PCR; the sequences of the primers used are provided in Table 1. The primers were designed using Primer Premier 5.0

### 2.3. Physiological and Biochemical Characteristics, and Antibiotic Susceptibility

The evaluation of physiological and biochemical traits was carried out utilizing standardized micro physiological and biochemical identification tubes. Antimicrobial susceptibility testing was executed in accordance with the Kirby-Bauer disk diffusion technique (Hangzhou Microbial Reagents Co., Ltd., Hangzhou, China).

### 2.4. Bacterial Challenge and Histopathological Analysis

A total of 200 fish were utilized in the experiment designed to determine the bacterial LD50 and to acquire samples for histopathological evaluation. A bacterial suspension of strain SCGY-1L was created through ten-fold serial dilutions, ranging from 2.74 × 10^1^ to 1.82 × 10^7^ CFU/mL. For the evaluation of pathogenicity, 180 healthy *S. chuatsi* were randomly assigned and allocated into six experimental groups (*n* = 30 per group). Each group of fish received intraperitoneal injections of 100 µL of the bacterial suspension at varying concentrations, while the control group was administered PBS. Cumulative mortality was monitored over a period of 7 days, and the LD_50_ value was computed using a modified arithmetic approach as described by Reed and Muench. For the histopathological examination, twenty fish were exposed to a 0.1 LD_50_ dose of the bacterial suspension, with samples being collected when the fish reached the moribund stage, just prior to death. Tissues were subsequently fixed in 10% neutral buffered formalin, embedded in paraffin, sectioned, and stained using hematoxylin and eosin (H&E). Prior to sample collection, the fish were anesthetized with tricaine methanesulfonate (MS222; Sigma, Beijing, China). All animal experiments adhered to the ARRIVE guidelines and received approval from the Animal Ethics Committee of Yangzhou University, under approval number 202503011 (1 March 2025).

### 2.5. Bacterial Library Construction and Genome Sequencing

Genomic DNA was extracted using a column-type bacterial genomic DNA extraction kit (TIANGEN BIOTECH (Beijing) Co., Ltd., Beijing, China) prior to sequencing. Genomic DNA was sequenced using a combination of PacBio Sequel IIe and Illumina sequencing platforms. For Illumina sequencing, genomic DNA was used for each strain in sequencing library construction. DNA samples were sheared into 400–500 bp fragments using a Covaris M220 Focused Acoustic Shearer following manufacture’s protocol. Illumina sequencing libraries were prepared from the sheared fragments using the NEXTFLEX Rapid DNA-Seq Kit (PerkinElmer, Inc., San Jose, CA, USA). Briefly, 5′ prime ends were first end-repaired and phosphorylated. Next, the 3′ ends were A-tailed and ligated to sequencing adapters. The third step is to enrich the adapters-ligated products using PCR. The prepared libraries then were used for paired-end Illumina sequencing (2 × 150 bp) on Illumina Novaseq 6000 (Illumina Inc., San Diego, CA, USA). For PacBio sequencing, genomic DNA was fragmented at ~10kb. DNA fragments were then purified, end-repaired and ligated with SMRT bell sequencing adapters following manufacturer’s recommendations (Pacific Biosciences of California, Inc., Menlo Park, CA, USA). Next, the PacBio library was prepared and sequenced on one SMRT cell using standard methods.

### 2.6. Genome Assembly and Gene Annotation

The data generated from PacBio Sequel IIe and Illumina platform were used for bioinformatics analysis. All of the analyses were performed using the free online platform of Majorbio Cloud Platform (http://cloud.majorbio.com (accessed on 10 May 2025)) from Shanghai Majorbio Bio-pharm Technology Co., Ltd. (Shanghai, China). The detailed procedures are as follows. The raw Illumina sequencing reads generated from the paired-end library were subjected to quality-filtered using fastp v0.23.0. The HiFi reads generated from the PacBio platform for analysis. Then the clean short reads and HiFi reads were assembled to construct complete genomes using Unicycle v0.4.8 and uses Pilon v1.22 to polish the assembly using short-read alignments, reducing the rate of small errors. The coding sequences of chromosome and plasmid were predicted using Prodigal and GeneMarkS, respectively. tRNA-scan-SE (v2.0) was used for tRNA prediction and Barrnap v0.9 (https://github.com/tseemann/barrnap (accessed on 10 May 2025)) was used for rRNA prediction. The predicted CDS were annotated from NR, Swiss-Prot, Pfam, GO, COG, KEGG and CAZY database using sequence alignment tools such as BLAST, Diamond and HMMER. Briefly, each set of query proteins were aligned with the databases, and annotations of best-matched subjects (e-value < 10^−5^) were obtained for gene annotation. Biosynthetic gene clusters (BGCs) of secondary metabolites were identified by antiSMASH v5.1.2 software.

### 2.7. Construction of Genome Circle Diagrams and Gene Functional Analysis

In this study, IslandViewer was utilized for the prediction of genomic islands, while Phigaro was employed for the prediction of prophages. For the prediction of CRISPR-Cas systems, Minced was used, and integron analyses were conducted using the Integron_Finder software v2.0.6. Genome circle diagrams were produced using both CGView v0.69-9 and Circos software v0.69. The assessment of metabolic functions, virulence factors, and resistance mechanisms was performed using the Carbohydrate-Active enZYmes Database (CAZy, http://www.cazy.org/ (accessed on 10 May 2025)), the Virulence Factor Database (VFDB, http://www.mgc.ac.cn/VFs/ (accessed on 10 May 2025)), and the Comprehensive Antibiotic Resistance Database (CARD, Version 1.1.3, https://card.mcmaster.ca/ (accessed on 10 May 2025)). Additionally, resistance gene analysis was carried out using the ResFinder database (https://genepi.food.dtu.dk/resfinder (accessed on 10 May 2025)).

### 2.8. Transcriptomic Analysis of Hosts Infected by Bacteria

The transcriptomic sequencing and subsequent analysis were executed by OE Biotech Co., Ltd., located in Shanghai, China. A total of 60 fish specimens were utilized for the preparation of transcriptomic sequencing samples. Following random assignment, the experimental cohort was exposed to a bacterial concentration of 0.1 × LD_50_, while the control cohort was administered injections of PBS solution. Tissues from every five fish were pooled into one homogenate tube per biological sample, with three parallel biological replicates collected for each experimental treatment. Transcriptomic analysis was conducted on the hepatic tissues of mandarin fish (*S. chuatsi*) that had been infected with the isolated strain SCGY-1L, with liver tissues from PBS-injected fish serving as control samples. Total RNA extraction was performed using TRIzol reagent in accordance with the manufacturer’s instructions. The sequencing libraries were processed on the Illumina Novaseq 6000 platform, generating paired-end reads of 150 bp in length. The raw reads in FASTQ format underwent preprocessing using fastp (length less than 50bp) to eliminate low-quality sequences, resulting in clean reads for further analysis. These clean reads were subsequently aligned to the reference genome (GCF_020085105.1) utilizing HISAT2 v2.2.1, followed by quantification of gene expression (FPKM) and read counts per gene obtained through HTSeq-count. Principal Component Analysis (PCA) and visualization were carried out utilizing R (v3.2.0) to evaluate the consistency of biological replicates. Differential expression analysis was performed using DESeq2 v1.34.0, which identified differentially expressed genes (DEGs) based on the criteria of q < 0.05 and |fold change| > 2.

### 2.9. Functional Annotation of Differentially Expressed Genes

Differentially expressed genes (DEGs) underwent functional enrichment analysis encompassing Gene Ontology (GO), KEGG pathways, Reactome, and WikiPathways. The number of differentially expressed genes (DEGs) belonging to each GO term was counted, and the significance of their enrichment was calculated using the hypergeometric test, yielding a *p*-value that indicates the enrichment significance. For pathway analysis, KEGG-based annotation of differentially expressed protein-coding genes was performed, followed by application of the hypergeometric test to evaluate the enrichment significance of these genes in each pathway. The results of the enrichment analysis were visualized through bar plots, chord diagrams, or enrichment circle plots (R v3.2.0). Gene Set Enrichment Analysis (GSEA) assessed predefined gene sets by ranking genes according to their differential expression levels between groups and testing for significant enrichment at the extremes of the ranked list.

### 2.10. Real-Time Quantitative PCR

Ten DEGs were randomly selected to analyze their expression levels using real-time quantitative PCR (RT-qPCR) on the remaining cDNA samples from transcriptome sequencing. The expression patterns of the selected genes were detected using SupRealQ Ultra Hunter SYBR qPCR Master Mix and an Applied Biosystems StepOnePlus Real-Time PCR System (ABI, Los Angeles, CA, USA). Each assay was performed in triplicate, with the expression of the β-actin gene serving as an internal control. Gene-specific primers are listed in Table S1. The qRT-PCR reaction was carried out in a 20 μL volume containing 10 μL of SYBR qPCR Master Mix (2×, Novizan), 1 μL of diluted cDNA, 0.5 μL each of forward and reverse primers, and 8 μL of RNase-free H_2_O. The reaction protocol was as follows: 95 °C for 5 min, followed by 40 cycles of 95 °C for 30 s, 60 °C for 15 s, and 72 °C for 10 s, with a final melt curve analysis from 72 to 95 °C. The results were calculated using the 2^−ΔΔCt^ method.

## 3. Results

### 3.1. Isolation and Identification of C. braakii SCGY-1L Strain

**Figure 1:**
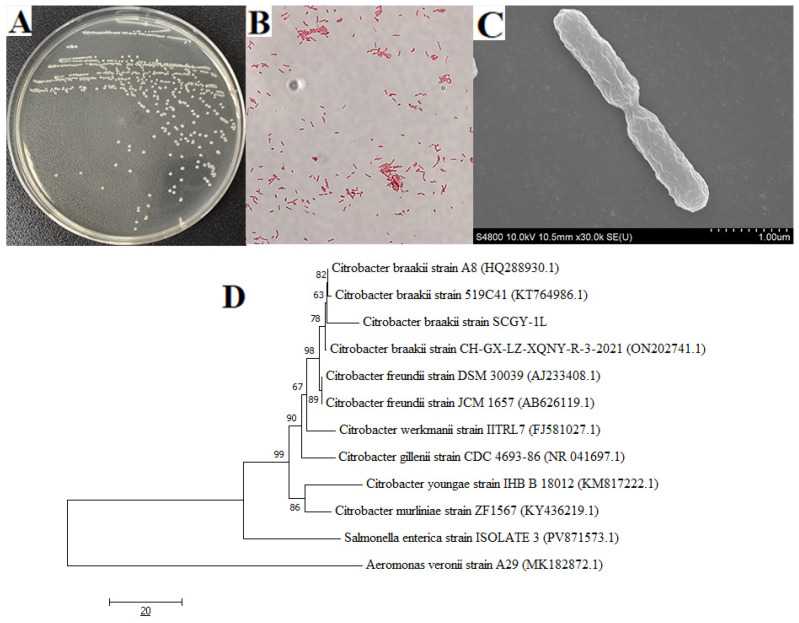
Isolation and identification of *C. braakii* SCGY-1L. (**A**) Colony morphology in LB agar. (**B**) Gram straining of *C. braakii* SCGY-1L observed by light micrograph; (**C**) Scanning electron microscope observation; and (**D**) Phylogenetic trees according to 16S rRNA sequence.

The target strain isolated from diseased fish liver tissues formed pinpoint, circular colonies on LB agar, exhibiting homogeneous circular colonies with smooth, moist, and translucent appearance (Figure 1A). Gram staining revealed Gram-negative bacilli (Figure 1B), while scanning electron microscopy confirmed rod-shaped bacterial cells (Figure 1C). Phylogenetic analysis based on 16S rRNA sequences demonstrated that the isolate clustered with known *C. braakii* strain (Figure 1D). Based on collective evidence, this bacterium was identified as *C. braakii* strain SCGY-1L. Physiological and biochemical characterization revealed that strain SCGY-1L was negative for aeculin, V-P test, indole, lysine decarboxylase and urea. Conversely, it demonstrated positive reactions for glucose gas, citrate, arginine, alanine, lactose, sucrose, maltose, sorbose, mannitol, raffinose, KCN, MR, nitrate reduction and ornithine decarboxylase. This biochemical profile aligns with *C. braakii* (Table 2).

### 3.2. Pathogenicity Analysis and Antibiotic Susceptibility Test

**Figure 2:**
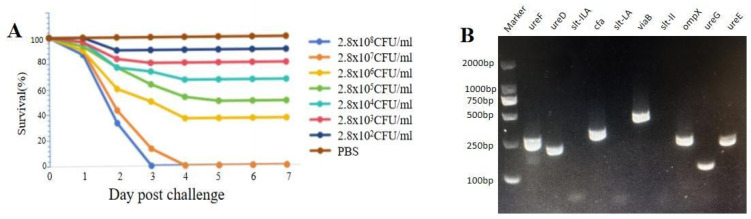
(**A**) Survival curves of *S. chuatsi* after *C. braakii* infection at diverse concentrations. PBS at an equivalent amount was injected in control group. (**B**) Agarose gel electrophoresis of the amplification products of virulent genes (*ureF*, *ureD*, *slt-ILA*, *cfa*, *slt-LA*, *viaB*, *slt-II*, *ompX*, *ureG*, *ureE*).

In the artificial infection trial, infected *S. chuatsi* initially showed no observable clinical signs; however, cumulative mortality occurred during the 7-day observation period, whereas control groups maintained 100% survival (Figure 2A). The LD_50_ of *C. braakii* SCGY-1L was found to be 1.28 × 10^6^ CFU/mL, with bacterial re-isolation from moribund specimens confirming biochemical and 16S rRNA genetic identity to the original *C. braakii* SCGY-1L isolate, thereby satisfying Koch’s postulates for pathogenicity. Virulence gene profiling further revealed seven critical determinants (*cfa*, *viaB*, *ompX*, *ureD*, *ureE*, *ureF*, *ureG*) through randomized PCR screening (Figure 2B). Concurrent antimicrobial susceptibility testing demonstrated that strain *C. braakii* SCGY-1L exhibited intermediate susceptibility to neomycin, minocycline, ampicillin, and cefradine, and resistance to erythromycin, medemycin, clindamycin, penicillin, oxacillin, and vancomycin, as quantitatively documented in Table 3.

### 3.3. Histopathology

**Figure 3:**
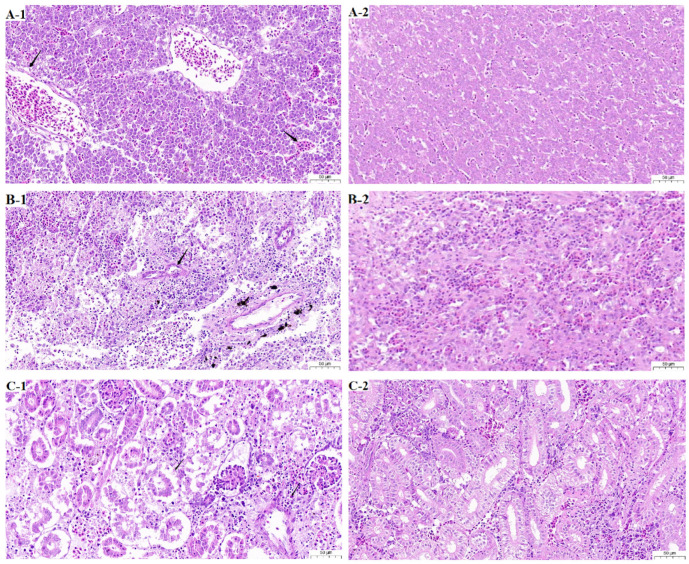
Haematoxylin and eosin staining of the tissues of *S. chuatsi* infected with *C. braakii*. (**A-1**) infected liver; (**A-2**) non-infected liver; (**B-1**) infected spleen; (**B-2**) non-infected spleen; (**C-1**) infected kidney; (**C-2**) non-infected kidney.

Obvious histopathological changes were observed in the moribund fish challenged with *C. braakii* SCGY-1L (Figure 3). Compared to the control group, fish infected with pathogenic bacteria exhibited microscopic, water-clearing vacuoles within the cytoplasm of hepatocytes, slight congestion was visible in the central veins and surrounding hepatic sinusoids (Figure 3A-1); kidney tissue displayed fewer renal tubules, with numerous renal tubular epithelial cells showing necrosis, sloughing, pyknotic and fragmented nuclei, and indistinct structures; spleen tissue revealed marked cellular necrosis.

### 3.4. Data Statistics and Genome Assessment

The whole-genome sequencing of isolate SCGY-1L revealed a complete genome size of 5,745,434 bp, while subsequent genomic annotation revealed 5482 coding genes with an average length of 920.32 bp, 8 rRNA operons and 86 tRNAs. By applying statistical methods to analyze the base distribution and quality fluctuations at each cycle of all sequencing reads, it is possible to macroscopically and intuitively reflect the library construction quality and sequencing performance: post-quality control base composition profiles are presented in Figure S1A, followed by the base error rate distribution after filtering in Figure S1B; Figure S1C displays per-base average quality scores across all sequencing reads, where the first half illustrates quality metrics for initial reads in paired-end sequencing and the latter half represents corresponding reads from the opposite ends; with Figure S1D subsequently showing the clean read length distribution.

### 3.5. Bacterial Gene Annotation

**Figure 4:**
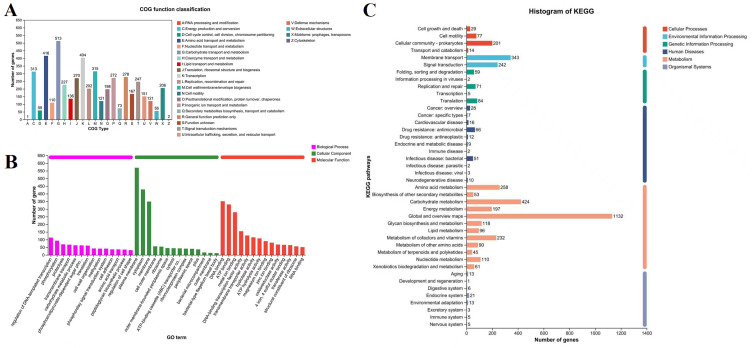
Functional annotation profiles of *C. braakii* SCGY-1L coding genes. (**A**) COG functional classification; (**B**) Gene Ontology (GO) term enrichment; (**C**) Histogram of KEGG.

Based on alignment results, functional annotation was performed using the NR, Swiss-Prot, Pfam, COG, GO, and KEGG databases. A total of 5431 genes were annotated in NR, 4516 in Swiss-Prot, and 4710 in Pfam. For strain SCGY-1L, 4324 genes (78.88% of total genes) were assigned to COG categories, with functional classification revealing metabolism-related genes as the most abundant category (37.58%), followed by cellular processes and signaling genes (26.92%), reflecting functional diversity essential for bacterial survival (Figure 4A). GO annotation assigned 3239 genes (59.08% of total) to functional categories (Figure 4B), broadly classified into cellular components, molecular functions, and biological processes. Molecular function annotations predominated, primarily enriched in DNA binding and ATP binding. KEGG pathway annotation identified 4111 genes (74.99%) mapped to metabolic pathways (Figure 4C), where metabolism constituted the most prominent level-1 functional category, followed by environmental information processing and cellular processes.

### 3.6. Bacterial Genomic Circos Plot Analysis

**Figure 5:**
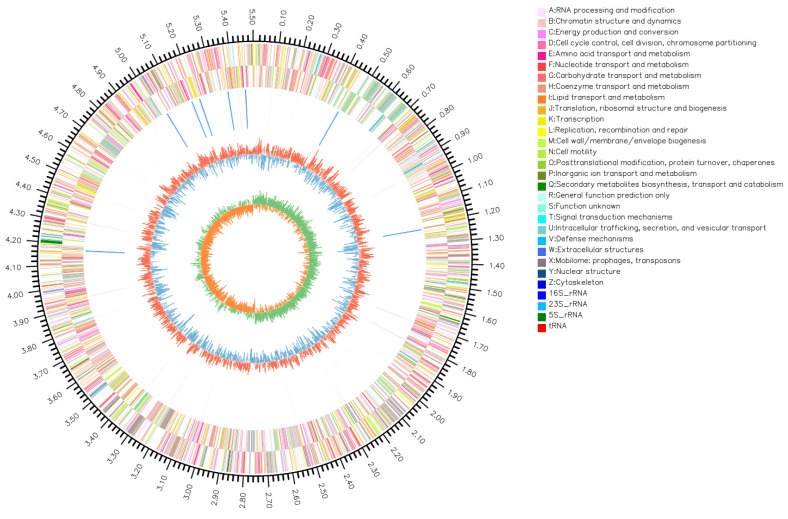
Genomic Architecture Visualization of *C. braakii* SCGY-1L via Circos Plot. The outermost ring indicates the genomic scale. The second and third rings represent coding sequences (CDS) on the forward and reverse strands, respectively, with colors designating COG functional categories. The fourth ring shows the locations of rRNA and tRNA. The fifth ring illustrates the GC content: outward red peaks indicate regions where the GC content is higher than the genomic average, while inward blue peaks represent regions with lower GC content. The innermost ring displays the GC skew value: values greater than 0 are shown in green and extend outward, whereas values less than 0 are colored yellow and extend inward.

The Circos plot comprehensively illustrates the genomic features of strain SCGY-1L, as depicted in Figure 5, revealing that SCGY-1L comprises one circular chromosome (Figure 5) and two plasmids (Figure S2A,B). Specifically, the chromosome measures 5,506,784 bp with a GC content of 51.91%, while plasmid P1 is 128,831 bp (51.35% GC) and plasmid P2 spans 109,819 bp (50.16% GC). Gene prediction analysis identified 5482 coding genes, with 5214 (95.11%) located on the chromosome, while plasmid P1 carries 145 genes and plasmid P2 harbors 123 genes, collectively accounting for 268 genes (4.89%) across both plasmids.

### 3.7. Carbohydrate-Active Enzymes Analysis

**Figure 6:**
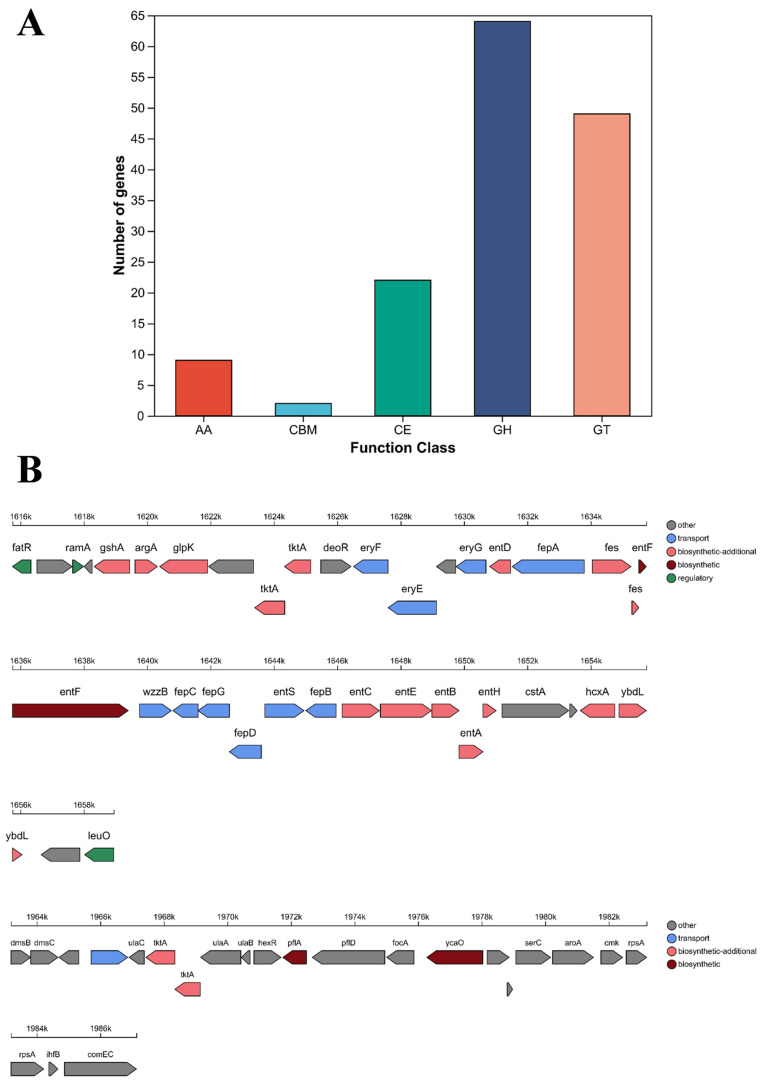
Functional Annotation of Carbohydrate-Active Enzymes and Secondary Metabolite Clusters. (**A**) Statistical Chart of CAZyme Annotations; (**B**) Linear map of biosynthetic gene clusters for secondary metabolites. The linear map displays all genes within the predicted gene cluster, where the colors of annotated genes correspond to their respective COG classifications. The functional categories represented by each color are detailed in the figure annotations, with gray indicating genes without COG annotation.

A total of 146 genes were annotated as carbohydrate-active enzymes (CAZymes), belonging to Glycoside Hydrolases (GHs), GlycosylTransferases (GTs), Carbohydrate Esterases (CEs), Carbohydrate-Binding Modules (CBMs), and Auxiliary Activities (AAs). Among these, GHs constituted the largest category with 64 genes, accounting for 52.17% of all CAZyme genes, followed by GTs with 49 genes (24.15%) (Figure 6A). The antiSMASH map revealed 38 genes annotated within an NRPS secondary metabolite gene cluster on the chromosome, located at genomic position 1,615,510–1,659,401 bp, while 22 genes were annotated within a thiopeptide metabolic gene cluster spanning position 1,961,742–1,988,033 bp on the chromosome (Figure 6B).

### 3.8. Pathogenicity-Associated Gene Analysis

**Figure 7:**
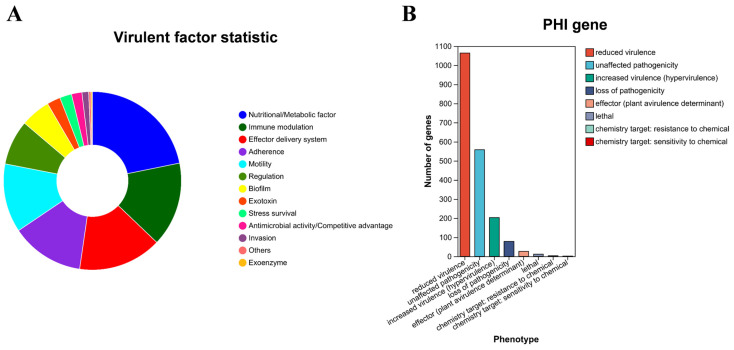
Genomic Virulence Profiling of *C. braakii* SCGY-1L. (**A**) Virulence factor classification by VFDB; (**B**) Pathogenic potential classification by PHI database.

This study predicted and analyzed pathogenicity-related genes through the Virulence Factor Database (VFDB) and Pathogen–Host Interactions (PHI) database (Figure 7). The VFDB analysis identified 738 virulence-associated genes, accounting for 13.46% of all genes, which were functionally categorized into: Nutritional/Metabolic factors (161 genes), Immune modulation (113 genes), Effector delivery system (112 genes), Adherence (98 genes), Motility (92 genes), Regulation (60 genes), and Biofilm formation (40 genes). Simultaneously, the PHI database predicted 1553 pathogenicity-related genes (28.33% of total genes), indicating the strain’s diverse pathogenic potential. These PHI genes were classified by mutant phenotype as: reduced virulence (1064 genes), unaffected pathogenicity (558 genes), increased virulence (203 genes), loss of pathogenicity (78 genes), effector genes (26 genes), including lethal factors (18 genes, 1.71%), and drug resistance genes (5 genes).

### 3.9. Antibiotic Resistance Gene Analysis

**Figure 8:**
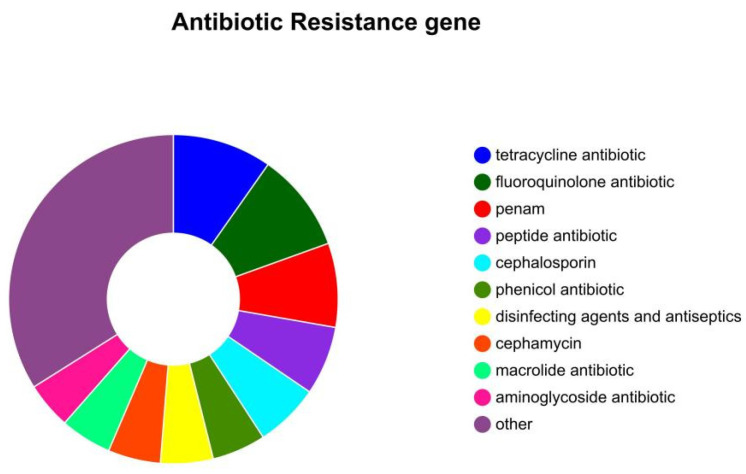
Genomic Antibiotic Resistome Profile of *C. braakii* SCGY-1L.

According to the CARD comparison, Figure 8 demonstrates the identification of 366 antibiotic resistance genes within the strain’s genome, with 362 located on the bacterial chromosome and 4 residing on plasmids. These genes confer resistance to tetracycline antibiotics (85 genes), fluoroquinolone antibiotics (85 genes), penams (72 genes), and peptide antibiotics (59 genes). Classification by resistance mechanisms revealed 231 genes associated with antibiotic efflux pumps, 94 genes linked to antibiotic target alteration, along with additional genes involved in antibiotic inactivation (19 genes), antibiotic target protection (17 genes), and reduced antibiotic permeability (13 genes) (Table S2).

### 3.10. Transcriptome Sequencing Data Statistics and Identification of Differentially Expressed Genes

**Figure 9:**
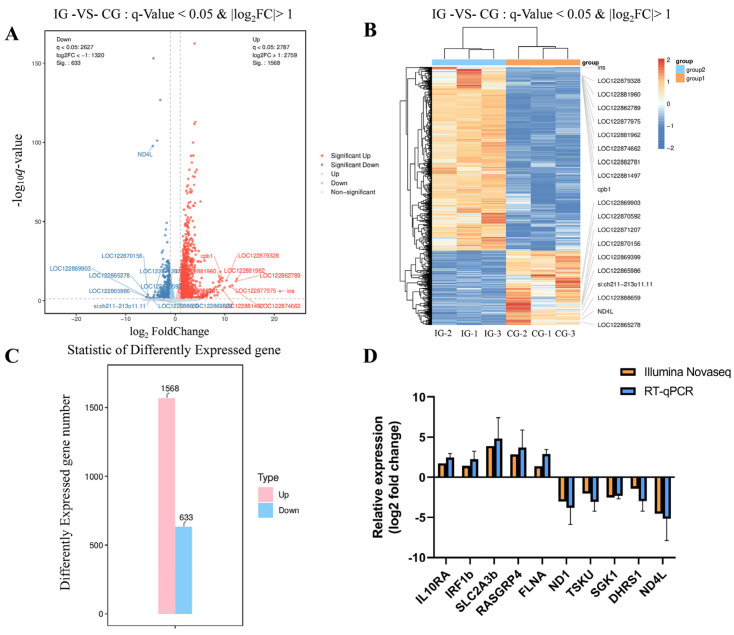
Differential gene expression profiling in *C. braakii* infection. (**A**) Volcano plot illustrating DEGs between IG and CG groups. (**B**) Heat map of increased and decreased DEGs. (**C**) Bar chart displaying the number of significant DEGs. (**D**) Comparison of the expressions of seven DEGs determined by Illumina Novaseq 6000 sequencing and RT-qPCR. The *x*-axis displays ten genes and *y*-axis is the relative expression level.

A total of 41.21 Gb of high-quality clean data were obtained from infected and control groups, with Q30 base ratios ranging from 95.74 to 96.07% and an average GC content of 48.63%, indicating robust sequencing quality. Reads were aligned to the reference genome, achieving alignment rates of 96.86–97.24% across samples, confirming high coverage and reliability. Gene expression levels were analyzed using scatter and heat maps (Figure 9A,B), and differentially expressed genes (DEGs) were filtered at thresholds of q < 0.05 and |log2FC| > 1. The analysis revealed significant transcriptional differences between groups: 2201 DEGs were identified (1568 upregulated and 633 downregulated; Figure 9C). The RT-qPCR results exhibited similar expression tendency as the high throughput sequencing data (Figure 9D). The qRT-PCR analysis confirmed the expressions of DEGs detected by the high-throughput sequencing analysis.

### 3.11. GO and KEGG Enrichment Analysis of Differentially Expressed Genes

**Figure 10:**
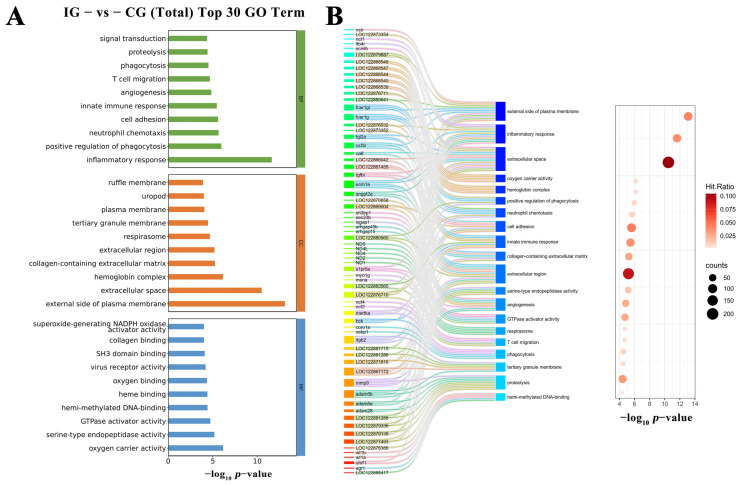
GO functional annotation of DEGs. (**A**) Bar plot showing the top 30 significantly enriched GO terms across the three categories, each with PopHits ≥ 5 and ranked by −log_10_(*p*-value). (**B**) Sankey and bubble plots illustrating the GO enrichment results. The Sankey plot highlights the relationship between DEGs and enriched GO terms. The bubble plot on the right visualizes the enrichment statistics: bubble size represents the number of DEGs involved in each GO term, while bubble color indicates the Hit Ratio, which is the proportion of DEGs among all genes associated with the corresponding GO term.

GO enrichment analysis identified 5229 GO terms, with 551 significantly enriched (*p*-value < 0.05). Terms meeting PopHits ≥ 5 were filtered, and the top 10 most significant entries (−log_10_ *p*-value) from each category—biological processes (BP), cellular components (CC), and molecular functions (MF)—were compiled into a top 30 enrichment result (Figure 10A). For BP, the top three terms were inflammatory response, positive regulation of phagocytosis, and neutrophil chemotaxis; for CC, external side of plasma membrane, extracellular space, and hemoglobin complex; for MF, oxygen carrier activity, serine-type endopeptidase activity, and GTPase activator activity. A Sankey bubble plot of the top 20 GO terms (ranked by *p*-value) illustrated relationships between DEGs and enriched functional categories (Figure 10B). Among these, four terms closely related to immunity—inflammatory response, innate immune response, phagocytosis, and immune response—are enriched with multiple key immune regulatory factors, such as hck, ptafr, ltb4r, and cxcr4b.

**Figure 11:**
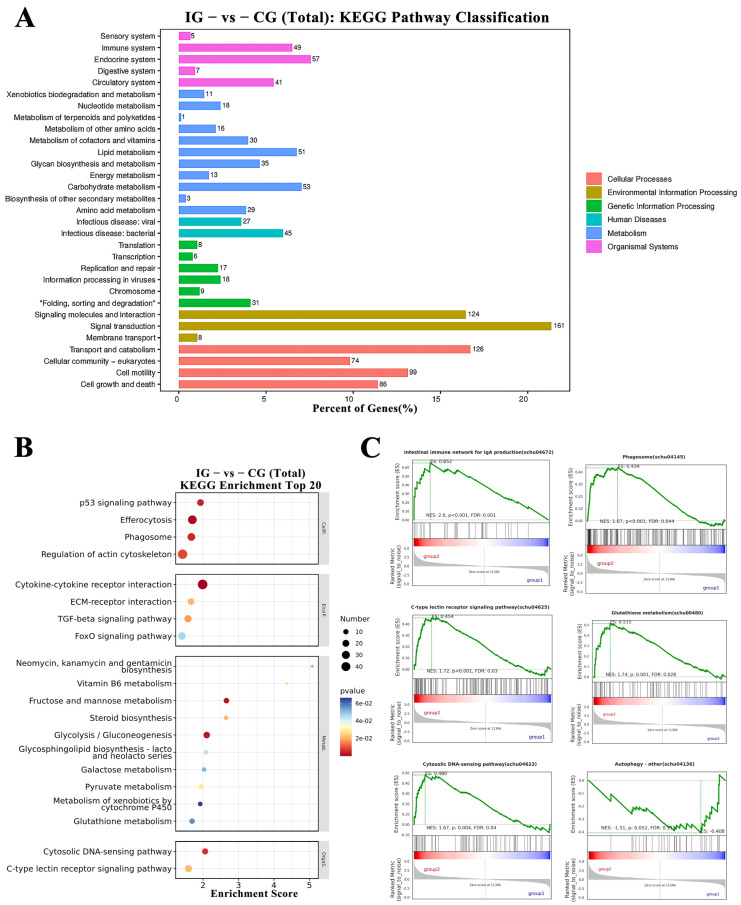
KEGG and GSEA Pathway Enrichment Analysis of DEGs. (**A**) KEGG Level 2 distribution of DEGs. (**B**) Top 20 KEGG pathway enrichment results presented as a bubble plot, ranked by −log_10_(*p*-value). The *x*-axis denotes the enrichment score, bubble size reflects the number of enriched DEGs per pathway, and bubble color ranges from blue to yellow to red, indicating increasing statistical significance. (**C**) GSEA plots of the six most enriched immune-related KEGG pathways. The core genes associated with each pathway are visualized. Pathways were considered significantly enriched based on the criteria: |NES| > 1, *p* < 0.05, and FDR < 0.25.

Integrated KEGG annotation and GSEA (Gene Set Enrichment Analysis) methodologies were employed for pathway enrichment studies of DEGs. As depicted in Figure 11A, KEGG Level 2 distribution revealed predominant DEG enrichment in Cellular Processes, Environmental Information Processing, and Metabolism. Pathways with PopHits ≥ 5 were filtered and ranked by −log_10_(*p*-value), generating a bubble plot of the top 20 enriched KEGG pathways (Figure 11B). Notably, Cytokine-cytokine receptor interaction, Efferocytosis, Phagosome, Cytosolic DNA-sensing pathway, Glutathione metabolism, and C-type lectin receptor signaling pathway exhibited significant enrichment, demonstrating activation of a complex immunoregulatory network during infection. To assess global enrichment trends, GSEA was performed on the top 10 upregulated and downregulated pathways. Figure 11C displays GSEA results for six immunity-linked pathways—Intestinal immune network for IgA production (schu04672), Glutathione metabolism (schu00480), C-type lectin receptor signaling pathway (schu04625), Phagosome (schu04145), Cytosolic DNA-sensing pathway (schu04623), and Autophagy—other (schu04136).

## 4. Discussion

*Citrobacter* spp. belong to the Enterobacteriaceae family, ubiquitously distributed in natural environments and commonly colonizing animal intestinal tracts. However, they can become pathogenic when host immunity is compromised [29]. While *Citrobacter* inhabits diverse natural hosts, this study isolated *C. braakii* from diseased mandarin fish. Subsequent whole-genome sequencing of this strain and transcriptomic profiling of infected hosts revealed the virulence determinants (including antibiotic resistance) of the *C. braakii* isolate SCGY-1L and its molecular modulation of immune defense pathways in mandarin fish, providing novel insights into the ecological transition of *Citrobacter* from commensalism to pathogenicity.

Current literature on *Citrobacter* isolation and foodborne transmission primarily focuses on *C. freundii* [30]. Multiple studies indicate its threat to both terrestrial and aquatic animals in intensive farming systems. Underreporting of *C. braakii* likely stems from frequent misidentification. High-resolution methods like whole-genome sequencing may increase detection accuracy, potentially revealing *C. braakii* as an emerging pathogen. To date, *C. braakii* has been detected in wastewater, meat products, ready-to-eat foods, animal-derived feeds, and fish [2]. Our infection experiments demonstrated that SCGY-1L could be re-isolated from hepatic tissues of moribund mandarin fish, with LD_50_ of 1.28 × 10^6^ CFU/mL. Histopathological analysis revealed significant alterations in hepatic, splenic, and renal tissues (Figure 2 and Figure 3), while control fish exhibited normal growth and no detectable abnormalities, confirming the high pathogenicity of *C. braakii* SCGY-1L in mandarin fish. In this study, the infection experiment employed intraperitoneal injection due to its well-recognized efficacy in eliciting a robust immune response. However, this infection method does not reflect the natural infection routes in aquaculture. We have acknowledged this limitation in the Discussion section, noting that the approach has certain constraints. Future work should further consider alternative exposure methods such as immersion and oral administration to better simulate natural infection pathways.

Tran et al. reported that the *C. braakii* ASE1 genome comprises a single circular chromosome of 5,021,820 bp with 52.2% GC content, encoding 4642 predicted coding sequences, 8 rRNA operons, and 83 tRNAs [31]. In contrast, SCGY-1L isolate in this study harbors 5482 protein-coding genes, 8 rRNA operons and 86 tRNAs. Schneider et al. isolated a strain of *C. braakii* from hospital wastewater, designated GW-Imi-1b1. By combining Illumina short-read sequencing and Nanopore long-read sequencing technologies, they successfully achieved a high-quality whole-genome assembly of the GW-Imi-1b1 strain. The genome of this strain consists of an approximately 5.1 Mb chromosome and 13 plasmids of varying sizes, demonstrating high genomic plasticity. It encodes a total of 5322 genes and shows 98.62% genomic similarity with *C. braakii* ASM207534v1 [32]. Although the number of plasmids identified in the SCGY-1L strain in this study is significantly fewer than that in GW-Imi-1b1, its predicted number of genes is greater. Further analysis revealed that SCGY-1L contains two operons, *vexABCDE* and *tviBCDE*. The tvi and vex operons encode the capsular polysaccharide Vi antigen, an important bacterial virulence factor, which may partly explain the significantly higher host mortality associated with this strain. Additionally, SCGY-1L carries multiple virulence factor genes related to motility and chemotaxis, such as pilus- and flagella-associated genes, as well as chemotactic signal transduction systems, indicating its potential pathogenicity. The presence of a type I pilus operon mediates host invasion, which is similar to the *fimAICDHFZYW* gene cluster in *Citrobacter freundii*, suggesting that *C. braakii* and *C. freundii* may have evolved from a common ancestor. However, the evolutionary mechanisms by which highly virulent or attenuated *C. braakii* strains cause severe infections in animals and humans warrant further investigation. Carbohydrate-Active Enzymes (CAZymes) were classified functionally into six major categories: Glycoside Hydrolases (GHs), Glycosyl Transferases (GTs), Carbohydrate-Binding Modules (CBMs), Carbohydrate Esterases (CEs), Auxiliary Activities (AAs), and Polysaccharide Lyases (PLs). Notably, CEs, GHs, and PLs are collectively termed cell-wall degrading enzymes (CWDEs) due to their role in decomposing plant cell walls during bacterial/fungal pathogenesis. As shown in Figure 6, 146 CAZyme genes (13.46% of total genes) were annotated in SCGY-1L, distributed across GHs, GTs, CEs, AAs, and CBMs, with no PLs detected. Further investigation of CAZymes is crucial for elucidating microbial carbohydrate metabolic mechanisms. Using the Virulence Factor Database (VFDB), 738 virulence-associated genes were identified (Figure 7), potentially explaining the high pathogenicity of this strain in mandarin fish.

Antibiotic misuse is particularly prevalent and severe in aquaculture, underscoring the critical need for antimicrobial susceptibility testing during bacterial disease outbreaks. Preliminary assessment of *Citrobacter* pathogenicity and drug sensitivity revealed resistance to multiple common antibiotics in our isolate (Table 3). This resistance profile is likely associated with antibiotic overuse, reflecting insufficient understanding of *Citrobacter* ecology and inadequate disease control measures. Furthermore, differential antibiotic resistance in *C. braakii* isolates from distinct hosts—potentially attributable to host specificity or geographic variation—may account for discrepancies between mandarin fish-derived *C. braakii* susceptibility data and other reports [33]. Genomic analysis via the Comprehensive Antibiotic Resistance Database (CARD) identified 366 resistance genes in the studied strain (Figure 8), with 362 chromosomally encoded and 4 plasmid-borne (Table S2). Carbapenem antibiotics represent the “last-line” treatment option for patients infected with antibiotic-resistant bacteria. According to the antimicrobial resistance report released by the World Health Organization (WHO), carbapenem-resistant Enterobacteriaceae are classified as a critical group of emerging resistant pathogens. Sun et al. demonstrated that *C. braakii* WF0082 co-harbors blaNDM-1, blaKPC-2, and mcr-9.1 on novel IncP6/IncX6 plasmids, facilitating the horizontal transfer of blaKPC-2 [34]. Wu et al. analyzed the genomes of *C. braakii* isolated from urban sewage and connected rivers, and the results indicated that these strains all belong to carbapenem-resistant *Citrobacter* [35], while SCGY-1L lacks genes such as blaKPC or blaNDM, suggesting that more extensive research and multifaceted data support are needed to evaluate the occurrence and transmission mechanisms of carbapenem resistance in *C. braakii*. A study on genomic data analysis of 20 food-derived *C. braakii* strains revealed that all strains carry the blaCMY gene encoding AmpC β-lactamase, exhibiting resistance to cephalosporins [8]. In contrast, SCGY-1L was not found to possess this gene, and drug susceptibility tests confirmed that this strain is sensitive to cephalosporin antibiotics. Furthermore, genomic prediction indicated that SCGY-1L carries a fluoroquinolone resistance gene; however, antimicrobial susceptibility testing demonstrated that the strain was sensitive to three quinolone antibiotics, revealing a discrepancy between the sequencing results and phenotypic susceptibility. We speculate that this fluoroquinolone resistance gene may remain silent due to promoter mutations or low efficiency of the ribosome binding site. Additionally, its expression might be environmentally dependent, requiring specific host signals or induction by subinhibitory concentrations of antibiotics, conditions not activated in standard in vitro susceptibility tests [36]. This precisely reflects the complexity of bacterial resistance evolution, and subsequent functional studies will be conducted to clarify whether this gene is silent, nonfunctional, or context-dependent. Furthermore, resistance to vancomycin in *C. braakii* is rarely reported, and the VanB gene present in SCGY-1L mediates vancomycin resistance, which is consistent with the drug susceptibility results of this study, indicating that this isolate demonstrates uniqueness in the spectrum of antimicrobial resistance genes. This suggests that *Citrobacter* species readily integrate exogenous resistance genes and may serve as reservoirs for multidrug resistance, necessitating enhanced clinical surveillance to prevent outbreaks. Consequently, during antibiotic use in aquaculture, bacterial resistance must be considered through rational drug selection to avoid antibiotic misuse and minimize resistance development. Additionally, strengthening husbandry management-including maintaining environmental hygiene and implementing regular disinfection-constitutes essential integrated control measures for preventing and controlling *C. braakii* infections.

The histopathological findings demonstrated that hepatic symptoms were most prominent in infected fish. As the liver is the primary organ for detoxification processes, biosynthesis, and cellular metabolism, transcriptomic analysis of *C. braakii*-infected mandarin fish elucidates mechanisms underlying hepatic regulation of inflammatory responses. Thus, liver samples were collected at 24 h post-infection for transcriptome sequencing. Both infected and control groups yielded 41.21 Gb of high-quality Clean Data, with alignment rates to the reference genome reaching 96.86–97.24%. While lower in data volume than Ding et al.’s report, the high genomic coverage ensures reliable alignment results [37]. Studies on transcriptomic analyses of *C. braakii* infecting other hosts are scarce, thus preventing us from conducting comparative discussions. Li et al. employed RNA-Seq technology to perform transcriptomic analysis on the hepatopancreatic tissues of *Procambarus clarkii*, investigating the impact of *C. freundii* infection on the metabolic functions and cellular health of the host hepatopancreas. Through KEGG pathway enrichment analysis of differentially expressed genes, they focused on alterations in lipid metabolism and cytochrome P450-related pathways. Their findings indicated that *C. freundii* infection significantly upregulates lipid degradation pathways and cytochrome P450-associated metabolic pathways, leading to reduced lipid content in hepatopancreatic cells, accumulation of reactive oxygen species (ROS), increased apoptosis, and ultimately causing structural damage to the hepatopancreatic tissue [38]. The pathway enrichment results differed between *C. braakii* infection in *S. chuatsi* and *C. freundii* infection in *P. clarkii*, in this study, Figure 10 presents GO and KEGG enrichment analyses of differentially expressed genes (DEGs), indicating that *C. braakii* infection triggers robust immune stress, signal modulation, and metabolic alterations in the host. Enrichment of genes (hck, ptafr, ltb4r, and cxcr4b) implies their core regulatory functions in immune cell activation, phagocytosis, and inflammatory cytokine release. DEGs were primarily enriched in three categories: Cellular Processes, Environmental Information Processing, and Metabolism, reflecting *C. braakii*-induced cellular functional responses and metabolic adjustments. Specifically, the Cytokine-cytokine receptor interaction, Glutathione metabolism, and C-type lectin receptor signaling pathway demonstrated significant enrichment (Figure 11B). The connection between the cytokine-cytokine receptor interaction pathway and bacterial virulence factors represents a critical battleground in host–pathogen interactions. This pathway serves as a foundation for regulating immune responses including inflammation, cell proliferation and differentiation, but is frequently targeted and manipulated by bacterial pathogens to enhance their survival and virulence. Recent studies indicate that bacteria directly interfere with host histone modifications through hemolysins and suppress pro-inflammatory cytokine expression by secreting effector proteins [39]. We speculate that the hemolysin gene of the SCGY-1L strain may also mediate this function, and the differential expression of host cytokines leads to altered host defense capabilities against this bacterial infection. The Glutathione metabolism pathway was also significantly enriched. Reduced glutathione (GSH), a tripeptide composed of glycine, glutamate, and cysteine, is widely present in most eukaryotes and some prokaryotes. Its primary functions include scavenging reactive oxygen species (ROS) to alleviate oxidative stress, and regulating protein function through S-glutathionylation modifications. Research has revealed that certain bacteria can utilize host GSH as a signaling molecule to activate their virulence gene expression. For instance, *Listeria* activates its virulence regulator PrfA through GSH [40], while *Burkholderia* activates the Type VI secretion system (T6SS) via GSH to promote intercellular spread [41]. SCGY-1L may regulate the host Glutathione metabolism pathway through its own virulence-related genes, thereby influencing its pathogenicity toward the host. C-type lectin receptors (CLRs) play crucial roles in host immune defense. As a class of pattern recognition receptors (PRRs) specialized in recognizing carbohydrate molecules, CLRs can identify glycosylated structures on the surfaces of both commensal and pathogenic bacteria, subsequently mediating immune cell activation and adaptive immune regulation. CLRs not only facilitate pathogen clearance but may also be exploited by certain bacteria to enhance their pathogenicity. Therefore, distinguishing between protective and pathogenic CLR-bacteria interactions is essential for developing novel preventive and therapeutic strategies. For example, epidermal Langerhans cells in the skin recognize specific glycosylated wall teichoic acids of *Staphylococcus aureus* and *Streptococcus pyogenes* through the CLR langerin, inducing local inflammatory responses and promoting pathogen clearance [42,43]. In contrast, Yersinia pestis utilizes CLR-mediated phagocytosis mechanisms to facilitate its own dissemination, demonstrating the dual nature of protection and pathogenesis in CLR-bacteria interactions [44]. In-depth understanding of the structural and functional relationships between CLRs and the glycosylated structures of the SCGY-1L strain may advance the prevention and treatment of infections mediated by this bacterium. Moreover, the significant enrichment of numerous other immune-relevant pathways suggests their critical roles in *C. braakii* infection immunity—though specific mechanisms require further investigation.

## 5. Conclusions

In this study, a bacterial strain was isolated from diseased *S. chuatsi* and identified as *C. braakii* through morphological characterization, physiological and biochemical tests, and molecular biological methods. The genomic sequencing and preliminary analysis of its pathogenicity and drug resistance were subsequently performed. Transcriptomic profiling of host responses to this bacterial infection was also conducted to elucidate immune responses. These results contribute to a better understanding of the immune response mechanisms against *C. braakii* infection and provide a theoretical reference for clinical diagnosis and treatment of diseases caused by this pathogen in *S. chuatsi*.

## Figures and Tables

**Table 1:** Primers for virulence gene detection.

Target Gene	Forward Primer (5′-3′)	Reverse Primer (5′-3′)	Reference or Genebank Accession No.
*cfa*	GCGGTTACTGGAAAGATG	CGGCGATACTGAAATAGG	CP137123.1
*viaB*	TGTCGAGCAGATGGATGAGCAT	ACGGCTGAAGGTTACGGACCGA	CP183865.1
*ompX*	CTACGAATACGGCTCTGC	ATCGGTTCCTTCACTTACAC	CP016762
*ureD*	GCCAGATGTCACGCATAACG	GGCTGCCACTGCTGATAGAA	CP128207.1
*ureE*	TAACAGGCTTTGGCGAGTAGGA	CGCCTTGACCACGCTCACT	CP069787.1
*ureF*	ATGCCGCAGAGTTGGCTGTC	GGAGATTGGCTGGGTGAAAA	CP174029.1
*ureG*	AGGTTATCGCCACCGCTTTC	GGTTGCCCGCATACTGCT	CP137175.1
*slt-II*	CCGGATCCATGAAGTGTATATTATTTAAATGG	CCCGAATTCTCAGTCATTATTAAACTGCAC	[27]
*slt-LA*	TTTACGATAGACTTCTCGAC	CACATATAAATTATTTCGCTC	[28]
*slt-ILA*	CCCGGATCCATGAAGTGTATATTATTTAAATGG	CCCGAATTCTTATTTACCCGTTGTATATAAAAA	[28]

**Table 2:** Physiological and biochemical features of isolates.

Characteristic	*C. braakii* SCGY-1L	Characteristic	SCGY-1L
Glucose gas	+	Sorbose	+
Citrate	+	Mannitol	+
Arginine	+	Raffinose	+
Alanine	+	KCN	+
Aesculin	−	MR	+
V-P test	−	H_2_S	+
Lactose	+	Nitrate reduction	+
Sucrose	+	ornithine decarboxylase	+
Indole	-	lysine decarboxylase	−
Maltose	+	Urea	−

Note: +, positive reaction; −, negative reaction.

**Table 3:** Antibiotic susceptibilities of *C. freundii* against 31 antimicrobial agents.

Antibiotic	Concentration (μg/piece)	Test Diameter of the Inhibition Zone (mm)	Sensitivity
Amikacin	30	18	I
Gentamicin	10	15	I
Kanamycin	30	20	S
Neomycin	30	16	I
Erythromycin	15	0	R
Medemycin	30	0	R
Norfloxacin	10	25	S
Ofloxacin	5	24	S
Ciprofloxacin	5	28	S
Polymyxin B	300	14	S
Clindamycin	2	0	R
Furazolidone	300	18	S
Tetracycline	30	20	S
Doxycycline	30	18	S
Minocycline	30	14	I
Penicillin	10	16	R
Oxacillin	1	0	R
Ampicillin	10	15	I
Carbenicillin	100	27	S
Piperacillin	100	22	S
Cephalexin	30	21	S
Cefazolin	30	26	S
Cefradine	30	18	I
Cefuroxim	30	22	S
Ceftazidime	30	21	S
Ceftriaxone	30	28	S
Cefoperazone	75	25	S
Vancomycin	30	0	R
Paediatric Compound Sulfamethoxazole Tablets	23.75/1.25	24	S
Chloramphenicol	30	27	S
Florfenicol	30	22	S

Note: S, susceptible; I, intermediate; R, resistant.

## Data Availability

The original contributions presented in the study are included in article/Supplementary Materials. The genome assembly generated in this study has been deposited in GenBank under the accession number JBQVYX000000000. The RNA seq Raw sequence reads have been deposited in the NCBI Sequence Read Archive (SRA) under the BioProject accession number PRJNA1321168.

## Supplementary Materials

The following supporting information can be downloaded at: https://www.mdpi.com/article/10.3390/microorganisms13102310/s1, Figure S1: Genomic sequencing quality assessment metrics of *C. braakii* Strain SCGY-1L; Figure S2: Genomic Architecture Visualization of the plasmid of *C. braakii* SCGY-1L via a Circos plot; Table S1: Primers used in RT-qPCR assay; Table S2: Comprehensive annotation of antibiotic resistance genes identified through CARD database screening.

## References

[1] Yao Y., Falgenhauer L., Falgenhauer J., Hauri A.M., Heinmüller P., Domann E., Chakraborty T., Imirzalioglu C. (2021). Carbapenem-Resistant *Citrobacter* spp. as an Emerging Concern in the Hospital-Setting: Results from a Genome-Based Regional Surveillance Study. Front. Cell Infect. Microbiol..

[2] Liu L., Zhang L., Zhou H., Yuan M., Hu D., Wang Y., Sun H., Xu J., Lan R. (2021). Antimicrobial Resistance and Molecular Characterization of *Citrobacter* spp. Causing Extraintestinal Infections. Front. Cell Infect. Microbiol..

[3] Zhang J., Qiao D., Wang H., Zhao X., Jiang X., Zhu L., Zhang J., Li L., Kong X., Pei C. (2025). Mixed Infection in Common Carp (*Cyprinus carpio*) Caused by *Aeromonas veronii*, *Aeromonas hydrophila*, *Plesiomonas shigelloides*, and *Citrobacter freundii*. Animals.

[4] Pastuszka A., Guz L., Puk K., Pietras-Ożga D. (2025). Occurrence of virulence factors and antimicrobial susceptibility of *Citrobacter freundii* isolated from diseased ornamental fish in Poland. J. Vet. Res..

[5] Nawaz M., Khan A.A., Khan S., Sung K., Steele R. (2008). Isolation and characterization of tetracycline-resistant *Citrobacter* spp. from catfish. Food Microbiol..

[6] Vega-Manriquez D.X., Davila-Arrellano R.P., Eslava-Campos C.A., Salazar Jimenez E., Negrete-Philippe A.C., Raigoza-Figueras R., Muñoz-Tenería F.A. (2018). Identification of bacteria present in ulcerative stomatitis lesions of captive sea turtles *Chelonia mydas*. Vet. Res. Commun..

[7] Huang C., Feng C., Liu X., Zhao R., Wang Z., Xi H., Ou H., Han W., Guo Z., Gu J. (2022). The Bacteriophage vB_CbrM_HP1 Protects Crucian Carp Against *Citrobacter braakii* Infection. Front. Vet. Sci..

[8] Pasquali F., Crippa C., Lucchi A., Francati S., Dindo M.L., Manfreda G. (2025). *Citrobacter braakii* Isolated from Salami and Soft Cheese: An Emerging Food Safety Hazard?. Foods.

[9] Dong X., Lv M., Zeng M., Chen X., Wang J., Liang X.F. (2025). Genome-Wide Identification, Characterization of the *ORA* (Olfactory Receptor Class A) Gene Family, and Potential Roles in Bile Acid and Pheromone Recognition in Mandarin Fish (*Siniperca chuatsi*). Cells.

[10] Zhu C., Li D., Chen W., Ban S., Liu T., Wen H., Jiang M. (2021). Effects of dietary host-associated *Lactococcus lactis* on growth performance, disease resistance, intestinal morphology and intestinal microbiota of mandarin fish (*Siniperca chuatsi*). Aquaculture.

[11] Zhang Z., Yuan X., Wu H., Gao J., Wu J., Xiong Z., Feng Z., Xie M., Li S., Xie Z. (2024). The effect of short-term artificial feed domestication on the expression of oxidative-stress-related genes and antioxidant capacity in the liver and Gill tissues of Mandarin Fish (*Siniperca chuatsi*). Genes.

[12] Zhang H., Zhou D., Dong J., Yan Y., Liu S., Ye X., He J., Sun C. (2025). Genomic Characterization and Pathogenicity of a Novel Birnavirus Strain Isolated from Mandarin Fish (*Siniperca chuatsi*). Genes.

[13] Zhou D.Y., Chen J.M., Sun Z.N., Zhang H.Q., Yuan M.Z., Wang X.B. (2025). Transcriptional landscape of brain adaptation to artificial feed domestication in mandarin fish (*Siniperca chuatsi*) revealed by weighted gene co-expression network analysis. Comp. Biochem. Physiol. Part D Genom. Proteom..

[14] He S., You J.J., Liang X.F., Zhang Z.L., Zhang Y.P. (2021). Transcriptome sequencing and metabolome analysis of food habits domestication from live prey fish to artificial diets in mandarin fish (*Siniperca chuatsi*). BMC Genom..

[15] Wang J.Y., Hao Y., Zhang L., Gao X., Xu Y., Wang J.J., Hanafiah F., Khor W., Sun Y., Wu C. (2024). Profiling the gut structure and microbiota, and identifying two dominant bacteria belonging to the weissella genus in mandarin fish (*Siniperca chuatsi*) fed an artificial diet. Front. Microbiol..

[16] Lu H.L., Li L., Miao Y.L., Liang H., Zou J.M., You J.J., Liang X.F., He S. (2023). Effects and regulatory pathway of proopinmelanocortin on feeding habit domestication in mandarin fish. Gene.

[17] Zeng Z.Y., Ding Z.L., Zhou A.N., Zhu C.B., Yang S., Fei H. (2024). Bacterial diseases in *Siniperca chuatsi*: Status and therapeutic strategies. Vet. Res. Commun..

[18] Rossi F., Santonicola S., Amadoro C., Marino L., Colavita G. (2023). Recent Records on Bacterial Opportunistic Infections via the Dietary Route. Microorganisms.

[19] He X., Wu J., Tan X., Xu S., Kong W., Liu X. (2025). Development of Duplex Loop-Mediated Isothermal Amplification with Hydroxynaphthol Blue for Detection of Infectious Spleen and Kidney Necrosis Virus and *Aeromonas hydrophila* in Chinese Perch (*Siniperca chuatsi*). Microorganisms.

[20] Gao J.H., Zhao J.L., Yao X.L., Tola T., Zheng J., Xue W.B., Wang D.W., Xing Y. (2024). Identification of antimicrobial peptide genes from transcriptomes in Mandarin fish (*Siniperca chuatsi*) and their response to infection with *Aeromonas hydrophila*. Fish Shellfish Immunol..

[21] Zhou W.D., Zhang Y.L., Wen Y., Ji W., Zhou Y., Ji Y.C., Liu X.L., Wang W.M., Asim M., Liang X.F. (2015). Analysis of the transcriptomic profilings of Mandarin fish (*Siniperca chuatsi*) infected with *Flavobacterium columnare* with an emphasis on immune responses. Fish Shellfish Immunol..

[22] Tian J.Y., Qi Z.T., Wu N., Chang M.X., Nie P. (2014). Complementary DNA sequences of the constant regions of T-cell antigen receptors α, β and γ in mandarin fish, *Siniperca chuatsi* Basilewsky, and their transcriptional changes after stimulation with *Flavobacterium columnare*. J. Fish Dis..

[23] Wu Y.L., Miao P.F., Yu H., Tan S.W., Yang H., Peng Z.Q., Yang Y. (2018). Isolation, identification and drug susceptibility test of pathogenic *Edwardsiella tarda* in *Siniperca chuatsi*. J. South. Agric..

[24] Zhu C.B., Shen Y.T., Ren C.H., Yang S., Fei H. (2024). A novel formula of herbal extracts regulates growth performance, antioxidant capacity, intestinal microbiota and resistance against *Aeromonas veronii* in largemouth bass (*Micropterus salmoides*). Aquaculture.

[25] Luo X., Fu X.Z., Liao G.L., Chang O.Q., Huang Z.B., Li N.Q. (2017). Isolation, pathogenicity and characterization of a novel bacterial pathogen *Streptococcus uberis* from diseased mandarin fish *Siniperca chuatsi*. Microb. Pathog..

[26] Weisburg W., Barns S., Pelletier D., Lane D. (1991). 16S ribosomal DNA amplification for phylogenetic study. J. Bacteriol..

[27] Mohammed M., Vignaud M.L., Cadel-Six S. (2019). Whole-Genome Sequences of Two Salmonella enterica Serovar Dublin Strains That Harbor the *viaA*, *viaB*, and *ompB* Loci of the Vi Antigen. Microbiol. Resour. Announc..

[28] Avijit R., Amer F., Barthe G., Brlansky R.H. (2005). A multiplex polymerase chain reaction method for reliable, sensitive and simultaneous detection of multiple viruses in citrus trees. J. Virol. Methods.

[29] Liu L.Y., Qin L.Y., Hao S., Lan R.T., Xu B.H., Guo Y.M., Jiang R.P., Sun H., Chen X.P., Lv X.C. (2020). Lineage, Antimicrobial Resistance and Virulence of *Citrobacter* spp.. Pathogens.

[30] Jiang G.L., Guo C.Y., Kuai X., Ji L.L., Zhao J., Zhou Y.X., Wang X.L., Zhang M.H., Zhou Z.M., Li H. (2025). Foodborne diarrhea outbreaks linked to *Citrobacter* species in East China 2022–2023. Food Microbiol..

[31] Tran T.D., Lee S., Hnasko R., McGarvey J.A. (2024). Complete genome sequence of *Citrobacter braakii* ASE1 generated by PacBio sequencing. Microbiol. Resour. Announc..

[32] Schneider D., Ganbarzade A., Post S., Zühlke D., Hinzke T., Hollensteiner J., Poehlein A., Riedel K., Daniel R. (2023). Complete Genome Sequence of *Citrobacter braakii* GW-Imi-1b1, Isolated from Hospital Wastewater in Greifswald, Germany. Microbiol. Resour. Announc..

[33] Oyeka M., Antony S. (2017). *Citrobacter braakii* Bacteremia: Case Report and Review of the Literature. Infect. Disord. Drug Targets.

[34] Sun F.Q., Yu X.M., Zhang F.L., Ma J., Ji P. (2025). Whole-genome sequencing of coexisting *bla*_NDM-1_, *bla*_KPC-2_, and *mcr-9.1* in a clinical carbapenem-resistant *Citrobacter braakii* isolate. J. Glob. Antimicrob. Resist..

[35] Wu T., Zou H., Xia H., Zhou Z., Zhao L., Meng M., Li Q., Guan Y., Li X. (2023). Genomic insight into transmission mechanisms of carbapenem-producing *Citrobacter* spp. isolates between the WWTP and connecting rivers. Ecotoxicol. Environ. Saf..

[36] Mohiuddin S.G., Kavousi P., Figueroa D., Ghosh S., Orman M.A. (2025). The diverse phenotypic and mutational landscape induced by fluoroquinolone treatment. mSystems.

[37] Ding W.D., Zhang X.H., Zhao X.M., Jing W., Cao Z.M., Li J., Huang Y., You X.X., Wang M., Shi Q. (2021). A Chromosome-Level Genome Assembly of the Mandarin Fish (*Siniperca chuatsi*). Front. Genet..

[38] Li M., Wang J., Deng H., Li L., Huang X., Chen D., Ouyang P., Geng Y., Yang S., Yin L. (2022). The Damage of the Crayfish (*Procambarus Clarkii*) Digestive Organs Caused by *Citrobacter Freundii* Is Associated with the Disturbance of Intestinal Microbiota and Disruption of Intestinal-Liver Axis Homeostasis. Front. Cell Infect. Microbiol..

[39] Denzer L., Schroten H., Schwerk C. (2020). From Gene to Protein—How Bacterial Virulence Factors Manipulate Host Gene Expression During Infection. Int. J. Mol. Sci..

[40] Mnich M.E., Dalen R.V., Sorge N.M.V. (2020). C-Type Lectin Receptors in Host Defense Against Bacterial Pathogens. Front. Cell. Infect. Microbiol..

[41] Wong J., Chen Y.H., Gan Y.H. (2015). Host cytosolic glutathione sensing by a membrane histidine kinase activates the type VI secretion system in an intracellular bacterium. Cell Host Microbe.

[42] Doebel T., Voisin B., Nagao K. (2017). Langerhans cells—The macrophage in dendritic cell clothing. Trends Immunol..

[43] Deckers J., Hammad H., Hoste E. (2018). Langerhans cells: Sensing the environment in health and disease. Front. Immunol..

[44] Yang K., Park C.G., Cheong C., Bulgheresi S., Zhang S., Zhang P., He Y., Jiang L., Huang H., Ding H. (2015). Host Langerin (CD207) is a receptor for Yersinia pestis phagocytosis and promotes dissemination. Immunol. Cell Biol..

