# Supplementary material for: Integrated Pathogen–Host Analysis of Citrobacter braakii SCGY-1L: Genomic Determinants and Host Transcriptional Dynamics During Infection
Source: Microorganisms. 2025 Oct 6;13(10):2310. doi: 10.3390/microorganisms13102310 (PMC12566268; doi:10.3390/microorganisms13102310)
Supplement: Supplementary file 1 [file microorganisms-13-02310-s001.zip › Supplementary Materials.pdf]

Table S1. Primers used in RT-qPCR assay.

Gene Symbol	Gene Name	Primer sequence 5′-3′	Gene ID
IL10RA	interleukin 10 receptor, alpha	F: ATACCTGAAAGATGATGTGGGG<br>R: GGTAGCACTAGACACTGTTTG	122879690
IRF1b	interferon regulatory factor 1b	F: GACCTCTGGAACAGCTTTTGT<br>R: AGCTTAAAGTGTGGACACCATC	122887997
SLC2A3b	solute carrier family 2 member 3b	F: CTTGAAAAGCTGGTTTTGGAGC<br>R: GTCTACTTAAGGCAACATGGC	122882042
RASGRP4	RAS guanyl releasing protein 4	F: CTACAAAGACAAGAGCAGAGAGCCA<br>R: CTGATGTGAATGGCCAGTACATCTC	122879077
FLNA	filamin A, alpha (actin binding protein 280)	F: GTTTGTCACGTCCTCAAAACAC<br>R: GAAAGATAGATTACGCACAAAGCG	122883096
ND1	NADH dehydrogenase subunit 1	F: GCCCTATTCTTCCTAGCAGAATAT<br>R: TTAGGTTGACTGCAGTGAGTTC	10965121
TSKU	tsukushi small leucine rich proteoglycan homolog	F: CGCACTGTGCTGTCATGTG<br>R: GGCCCCAAGCCTTTACAG	122878668
SGK1	serum/glucocorticoid regulated kinase 1	F: TAGAGAGAGAGAGAGAAAGAGAGAGC<br>R: TTGTGAGGAAGACTAAATGAGGGGG	122863241
DHRS1	dehydrogenase/reductase (SDR family) member 1	F: GGGAAGTGCATCGTCAATCT<br>R: TACAGTCCTTCCATCAACATCTC	122886786
ND4L	NADH dehydrogenase subunit 4L	F: CTTCTTACTAGGGTTAACAGGAC<br>R: TCCATAGGGAGAGTGCAATAAAC	10965117
β-actin	beta-actin (ACTb) gene	F: CCCTCTGAACCCCAAAGCCA<br>R: CAGCCTGGATGGCAACGTACA	FJ436084.1

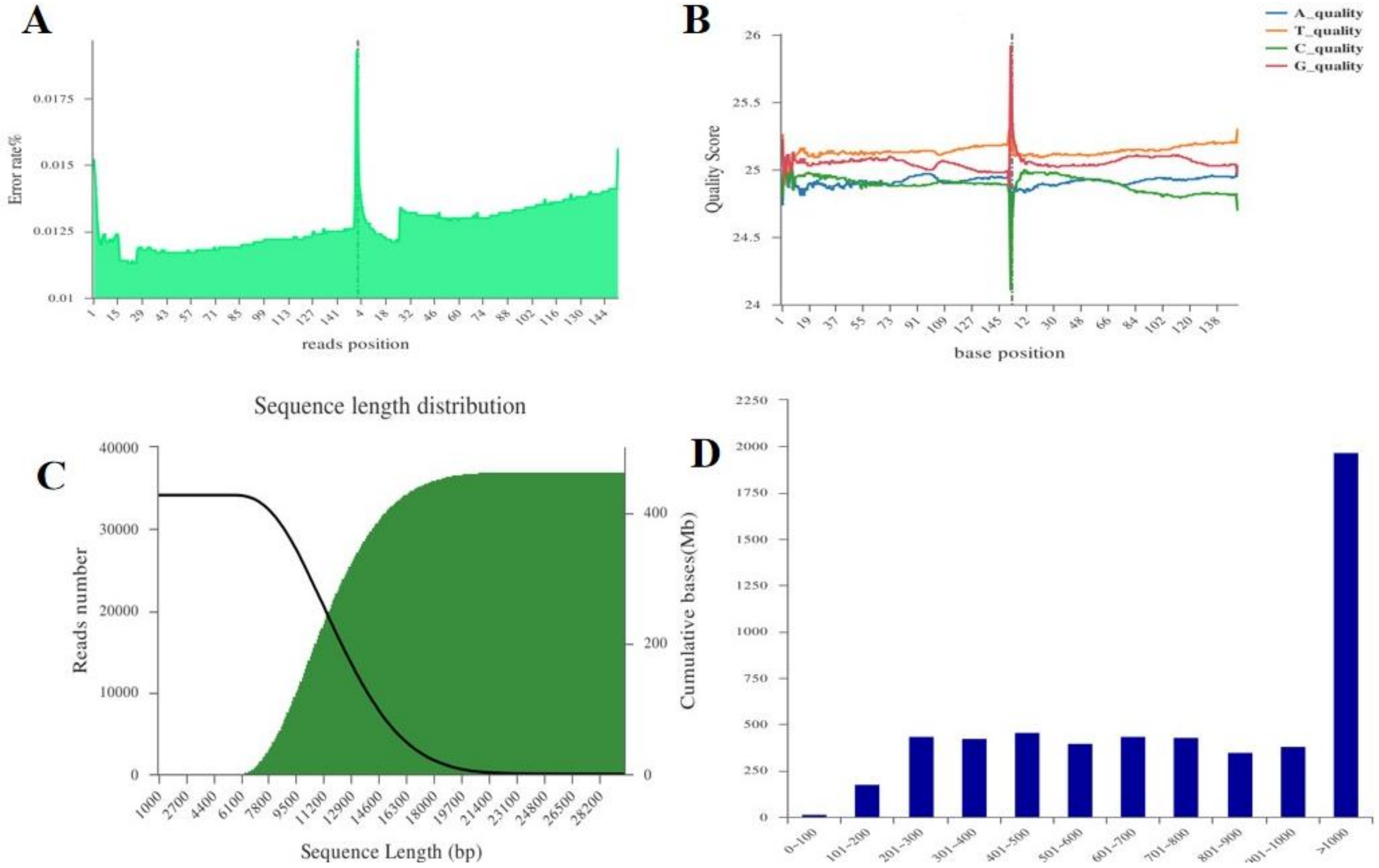

Figure S1. Genomic sequencing quality assessment metrics of *C. braakii* Strain SCGY-1L. (A) Error rate distribution per base position before quality control. X-axis: Base position (bp); Y-axis: Single-base error rate (%); (B) Base quality distribution of clean data summarized by base position. X-axis: Base position; Y-axis: Mean quality score (Q-score); (C) Clean reads length distribution from PacBio sequencing data. X-axis: Reads length (bp); Y-axis: Reads count; (D) Length distribution of protein-coding genes.X-axis: Gene length range (bp); Y-axis: Number of genes.

A

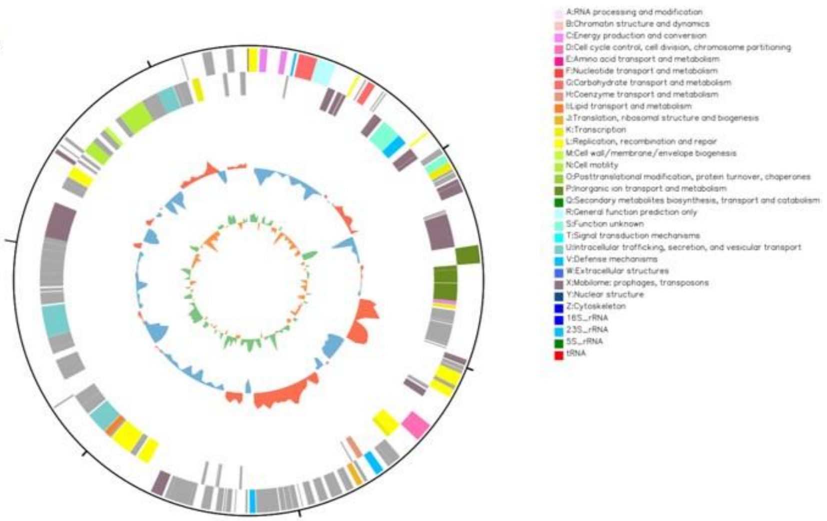

0.10
A:RNA processing and modification
B:Chromatin structure and dynamics
C:Energy production and conversion
D:Cell cycle control, cell division, chromosome partitioning
E:Amino acid transport and metabolism
F:Nucleotide transport and metabolism
G:Carbohydrate transport and metabolism
H:Coenzyme transport and metabolism
I:Lipid transport and metabolism
J:Translation, ribosomal structure and biogenesis
K:Transcription
L:Replication, recombination and repair
M:Cell wall/membrane/envelope biogenesis
N:Cell motility
O:Posttranslational modification, protein turnover, chaperones
P:Inorganic ion transport and metabolism
Q:Secondary metabolites biosynthesis, transport and catabolism
R:General function prediction only
S:Function unknown
T:Signal transduction mechanisms
U:Intracellular trafficking, secretion, and vesicular transport
V:Defense mechanisms
W:Extracellular structures
X:Mobilome: prophages, transposons
Y:Nuclear structure
Z:Cytoskeleton
16S_rRNA
23S_rRNA
5S_rRNA
tRNA

B

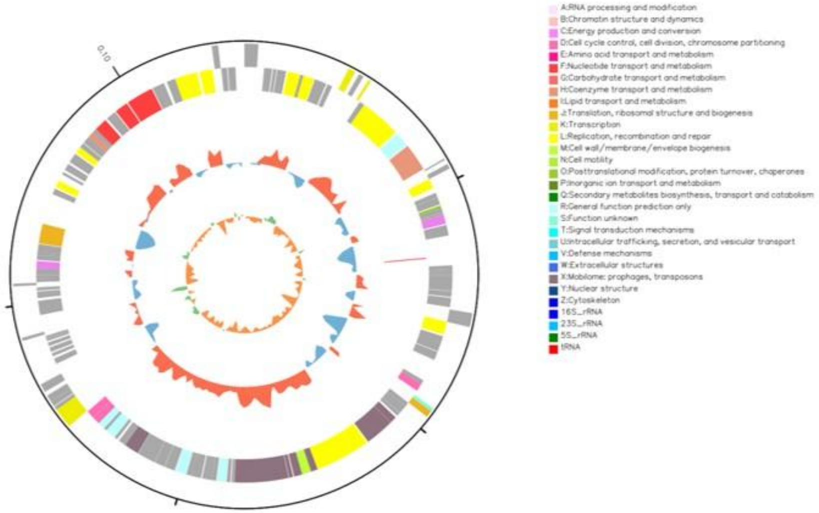

0.10
A:RNA processing and modification
B:Chromatin structure and dynamics
C:Energy production and conversion
D:Cell cycle control, cell division, chromosome partitioning
E:Amino acid transport and metabolism
F:Nucleotide transport and metabolism
G:Carbohydrate transport and metabolism
H:Coenzyme transport and metabolism
I:Lipid transport and metabolism
J:Translation, ribosomal structure and biogenesis
K:Transcription
L:Replication, recombination and repair
M:Cell wall/membrane/envelope biogenesis
N:Cell motility
O:Posttranslational modification, protein turnover, chaperones
P:Inorganic ion transport and metabolism
Q:Secondary metabolites biosynthesis, transport and catabolism
R:General function prediction only
S:Function unknown
T:Signal transduction mechanisms
U:Intracellular trafficking, secretion, and vesicular transport
V:Defense mechanisms
W:Extracellular structures
X:Mobilome: prophages, transposons
Y:Nuclear structure
Z:Cytoskeleton
16S_rRNA
23S_rRNA
5S_rRNA
tRNA

Figure S2. Genomic Architecture Visualization of the plasmid of *C. braakii* SCGY-1L via a Circos plot.The outermost ring indicates the genomic scale. The second and third rings represent coding sequences (CDS) on the forward and reverse strands, respectively, with colors designating COG functional categories. The fourth ring shows the locations of rRNA and tRNA. The fifth ring illustrates the GC content: outward red peaks indicate regions where the GC content is higher than the genomic average, while inward blue peaks represent regions with lower GC content. The innermost ring displays the GC skew value: values greater than 0 are shown in green and extend outward, whereas values less than 0 are colored yellow and extend inward.e. (A) Plasmid P1 structural overview; (B) Plasmid P2 structural overview.
